# A single center comparative study of two single use digital flexible ureteroscopy in the management of renal stones less than 2 cm

**DOI:** 10.1007/s00345-023-04290-6

**Published:** 2023-01-26

**Authors:** Fouad Zanaty, Mohamed Elshazly, Hossam Kandeel, Baher Salman

**Affiliations:** Department of Urology, Menoufia University Hospitals, Shibin el Kom, Egypt

**Keywords:** Single use flexible ureteroscopy, Renal stones, Pusen, LithoVue

## Abstract

**Purpose:**

For renal stones < 2 cm, guidelines recommend the use of retrograde intrarenal surgery as a first line treatment option. Many available single use flexible ureteroscopy were found. We aim to compare the effectiveness of two single use flexible ureteroscopy; Pusen Uscope 3011 versus LithoVue in the management of renal stones less than 2 cm.

**Methods:**

Our study prospectively included 60 patients equally divided in to two groups: Pusen group and LithoVue group during the period from June 2020 to June 2021. The included patients were above 18 years old. Perioperative details as operative time, fluoroscopy time, hospital stay, and complications were recorded. Stone free rate was assessed. Base purchase cost was also compared.

**Results:**

There was no statistically significant difference between the two groups regarding age, gender, and body mass index (BMI), stones size, side, number and location. The perioperative evaluation and outcome had no statistically significant differences between the two groups regarding the operative time, hospital stay, access sheath use, and stone free rate or radiation exposure. Among all cases, we had 49 cases (81.6%) with no postoperative complications (21 cases for Pusen group and 28 cases for LithoVue group). The incidence of postoperative complications was significantly higher among Pusen group than LithoVue group (*p* = 0.02). Initial purchase cost for both FURS had no significant difference (*P* = 0.86).

**Conclusion:**

RIRS can be performed effectively with Pusen 3011 and LithoVue single use flexible ureteroscopy in patients diagnosed with renal calculi < 2 cm with superior outcomes with LithoVue.

## Introduction

Urinary urolithiasis is one of the most common diseases in the urology field (approximately 12% of the world’s population). [1] For renal stones < 2 cm, guidelines from the European and American Urological Associations recommend the use of retrograde intrarenal surgery (RIRS) as a first line treatment option, alternative to extracorporeal shock wave lithotripsy (SWL) or percutaneous nephrolithotomy (PCNL) [2] [3].

In 1964, the endoscopic access to the renal collecting systems was firstly introduced [4] and with technical and optical improvements; RIRS has become a more widespread approach. Currently, there is a variety of flexible ureteroscopes (FURS) available, including reusable and disposable ureteroscopes and due to the high cost, limited durability and lack of need for sterilization; the disposable FURS have been developed to overcome some of the limitations of reusable FURS [5]. Also, only experienced hands can use reusable FURS perfectly without damage, on the other hand, single use FURS can be performed by juniors very easy so, advantages of single use FURS gaining more popularity [6].

Many single use FURS are found commercially. Pusen Uscope UE3011 (Zhuhai Pusen Medical Technology Co., Ltd. China) and LithoVue (Boston Scientific, Marlborough, MA) were the first digital single use FURS in the Egyptian market. The Pusen Uscope UE3011, has the same dimensions and deflection as the LithoVue, but is designed with a semiflexible shaft and a flexible tip only. Also, the design of the handle differs; the Pusen UE3011 provides a pistol-like handle [7]. In this study, we aimed to compare the effectiveness of Pusen Uscope 3011 versus LithoVue FURS in the management of renal stones less than 2 cm.

## Methods

This prospective study was carried out at Urology department, Menoufia university hospitals, Egypt during the period from June 2020 to June 2021. Sixty patients were included and were randomly divided into two groups: Pusen group and LithoVue group. The study protocol was approved from the Ethics Committee of our faculty and university. The included patients were above 18 years old with renal stones up to 2 cm. An informed consent was obtained from all patients. Patients with renal or skeletal anomaly, ureteral stone, chronic kidney disease, solitary kidney, stones in calyceal diverticulum and patient with coagulopathy were excluded from the study.

All patients were preoperatively evaluated by medical history, examination, routine pre-operative investigations including urine culture, pelvi-abdominol ultrasound, plain X ray (KUB) and spiral C.T scan. Preoperative antibiotic prophylaxis had given according to the culture and sensitivity test. After general anesthesia, the patient was placed in the dorsal lithotomy position. With cystoscopy, identification of ureteric orifice and a hydrophilic guide wire was introduced. Diagnostic semi-rigid ureteroscopy was done to assess the ureter. Next, 12 F / 45 cm ureteric access sheath (UAS) (Coloplast, PORGES, France) was placed through the wire under fluoroscopy guidance. Retrograde access to the upper urinary tract was obtained with one of both types of FURS. Once the calculus is seen, 200 µm laser fiber was inserted for lithotripsy. The lithotripsy stopped when small stone fragments (< 3 mm) were seen. Small stones and fragments were retrieved with a 1.9 F zero tip nitinol basket. A 5-6Fr/ 26–28 cm double J stent was inserted routinely at the end of the procedure for both prevention and treatment of ureteral obstruction or infection complications following FURS.

Perioperative details as operative time, fluoroscopy time, hospital stay, and complications were recorded. Stone free rate (SFR) was assessed by plan abdominal X-ray (KUB) and ultrasound after 1 week and by spiral CT scan after 2 months. Base purchase cost was also compared because it is an important factor in every country health system.

The data were collected, tabulated, and analyzed by SPSS (statistical package for social science) version 17.0 on IBM compatible computer (SPSS Inc., Chicago, IL, USA). Qualitative data were described as number and percentage. Shapiro–Wilk test was used to verify the normality of distribution. Quantitative data were expressed as mean and standard deviation and qualitative data was compared using chi square and Fisher's Exact test. Significance level was considered at 0.05.

## Results

The study included 30 patients in each group with mean age 45 years and average stone volume 13.5 ± 3.9 mm^3^ for Pusen group and mean age 43 years with average stone volume 13.3 ± 3.3 mm^3^ for LithoVue group. Most of the cases, 55 cases (91.7%), were with single stones. There was no statistically significant difference between the two groups regarding age, gender and body mass index (BMI), stones size, side, number and location. Detailed patient demographic parameters and stone characteristics were summarized in Table 1.

**Table 1:** Detailed patient demographic parameters and stone characteristics

Mean Age (years)	Pusen Group (*n* = 30)		LithoVue Group (*n* = 30)		*P* value
	45 ± 14 (31—59)		43 ± 13 (30—56)		0.6
Sex					
Male	12	20%	13	21.6%	0.5
Female	18	30%	17	28.4%	0.5
BMI (mean)	24.9 ± 5.5 (19.4 to 30.4)		24.7 ± 5 (19.7 to 29.5)		0.8
Stone Volume (average)	13.5 ± 3.9 mm^3^		13.3 ± 3.3 mm^3^		0.9
Hounsfield Units (HU)	1036 ± 340		1203 ± 310		0.065
Stone Side					
Right	12 (40%)		17 (56.7%)		0.1
Left	18 (60%)		13 (43.3%)		0.1
Stone number					
Single	27 (90%)		28 (93.3%)		0.1
Multiple	3 (10%)		2 (6.7%)		0.1
Stone site					
Upper	3 (10%)		4 (13.3%)		0.7
Middle	6 (20%)		6 ((20%)		0.7
Pelvis	9 (30%)		7 (23.4%)		0.7
Lower	12 (40%)		13 (43.3%)		0.7

As regard operative time, it was 90 ± 45.5 min in Pusen group and 89.2 ± 41.4 min in LithoVue group. Hospital stay was 36 ± 34.22 h in Pusen group and 34 ± 33.67 h for LithoVue group.

We had 20 cases stone free (66.7%), 7 cases with residual fragments (5–7 mm) (23.3%) and 3 cases with failed procedure (10%) in Pusen group but, in LithoVue group, we had 24 cases stone free (80%), 4 cases with residual fragment (13.3%) and 2 cases with failed procedure (6.7%). Stone free defined by no residual stone or insignificant residual stones ≤ 4 mm [8]. Regarding preoperative stenting, we had total 32 cases with prior stenting and 28 cases not stented pre-operatively. In Pusen group, 17 cases (56.7%) had prior stenting versus 15 cases (50%) stented preoperatively in LithoVue group. SFR had no significant difference between the two groups (*p* = 0.3) and preoperative stenting had also no significant difference (*p* = 0.6).

Among all cases we had 49 cases (81.6%) with no postoperative complications (21 cases for Pusen group and 28 cases for LithoVue group). There were six cases (10%) where patients suffered from post-operative fever (Clavien classification grade II) due to urinary tract infection (UTI), five cases of them in Pusen group versus one case in LithoVue group (*P* = 0.03). Mild hematuria (Clavien classification grade I) were found in three cases in Pusen group and one case in LithoVue group (*P* = 0.01). There is only one case with intraoperative ureteric mucosal injury (Clavien classification grade I) caused by UAS (*P* = 0.1). The perioperative evaluation and outcome are illustrated in Table 2 with no statistically significant differences between the two groups regarding the operative time, hospital stay, preoperative stenting, access sheath use, and SFR or radiation exposure but, the incidence of postoperative complications was significantly higher among Pusen group than LithoVue group (*p* < 0.05). Initial purchase cost was 875 US dollar for Pusen FURS versus 1000 US dollar for LithoVue FURS with no significant difference between the two groups (*P* = 0.86).

**Table 2:** Pri-operative evaluation and outcome

	Pusen group		LithoVue group		*P* value
	No	%	No	%	
Operative time (min)	90.7 ± 45.5	89.2 ± 41.4	0.8		
Access sheath					
Yes	23	76.7	21	70.0	0.559
No	7	23.3	9	30.0	
Radiation exposure (min)	5.5 ± 3	5.6 ± 3.2	0.9		
Preoperative stenting	17 (56.7%)	15 (50%)	0.6		
Hospital stay (hours)	36 ± 34.22	34 ± 33.67	0.86		
Stone free rate (SFR)	20	66.7	24		
Residual stone (5–7 mm)					
Lower calyx	4	13.3	4	13.3	
Middle calyx	3	10	0	0.0	
Failed procedure	3	10.0	2	6.7	1.000
Complications					
No	21	70	28	93.3	0.02
Fever (Clavien grade II)	5	16.7	1	3.3	0.03
Hematuria (Clavien grade I)	3	10	1	3.3	0.01
Ureteric injury (Clavien grade I)	1	3.3	0	0.0	0.1
Cost (US *Dollar)*	875	1000	0.86		

## Discussion

Since 2020, we managed our cases with renal stone < 2 cm with FURS instead of SWL or PCNL. In this study, there was no statistically significant difference between the two groups regarding age, gender and body mass index (BMI), stones size, side, number and location. The mean operation time was 90.7 ± 45.5 min for Pusen group and 89.2 ± 41.4 for LithoVue group, which little longer than the operation time in the study done by Guisti et al., 2016 (80 ± 35.5 min) [9]. It was due to our early experience in usage of single use FURS. Kuroda et al., 2018 also predict 6 causes of increase FURS operative time (preoperative stent, stone volume, maximum HFU, ureteral access sheath diameter, patient sex and lastly operator experience) [10].

Regarding stone free rate (SFR), we had 20 cases stone free (66.7%), 7 cases with residual fragments (5–7 mm) (23.3%) and 3 cases with failed procedure (10%) in Pusen group but, in LithoVue group, we had 24 cases stone free (80%), 4 cases with residual fragment (13.3%) and 2 cases with failed procedure (6.7%). In the study done by Ekici et al., 2019 the SFR was 67% after the first session of FURS and 90% after second session [11]. This was similar to our study as the stone free rate ranged from 67 to 80%. Other studies showed higher SFR than our study as Jessen et al., 2014 who reported about 88% stone free rate among 111 cases with calyceal stones [12]. We had 25 cases of lower calyx stones (41.7%); 68% were stone free and 32% with residual (< 4 mm), with no significant difference between the two groups. Our results were parallel to Xun et al., 2020 in which stone free rate in lower calyceal stones about 70% [13].

Portis et al., in 2014 concluded that any residual fragment may be associated with high risk of regrowth and retreatment [14] so, for residual stone (5–7 mm), retreatment with SWL was performed. For asymptomatic stones < 4 mm, we would not intervene. Intervention is indicated if there was evidence of persistent infection.

UAS was used successfully in 23 cases (76.7%) in Pusen group and in 20 cases (70%) in LithoVue group (*P* = 0.56). The use of ureteral access sheath (UAS) is useful to facilitate ureteric entry, decrease operative time, decrease intrarenal pressure which may decrease the postoperative UTI and less traumatism to the scopes [15]. Failed procedure was due to the complex renal anatomy or due to neglected DJ with severe peri-ureteritis, which make the ureter not malleable to introduce the FURS. Sanguedolce et al., 2017 concluded that the efficacy of RIRS depends on stone (composition, number, and size), anatomical site (e.g., lower pole) and surgeon (volume/experience) characteristics [16]. PCNL was done for non-accessible stones.

Among all cases, we had 49 cases (81.6%) with no postoperative complications (21 cases for Pusen group (A) and 28 cases for LithoVue group). There were six cases (10%) suffered from post-operative fever (grade II) due to urinary tract infection (UTI), five cases of them in Pusen group versus one case in LithoVue group (*p* = 0.03) (Table 2). All cases managed conservatively by fluids, antibiotics and antipyretics with no serious outcomes. Postoperative hematuria (grade I) were found in four cases (6.7%), three cases in Pusen group and one case in LithoVue group (*P* = 0.01). It was managed conservatively with fluids and hemostatic agents.

**Fig. 1:**
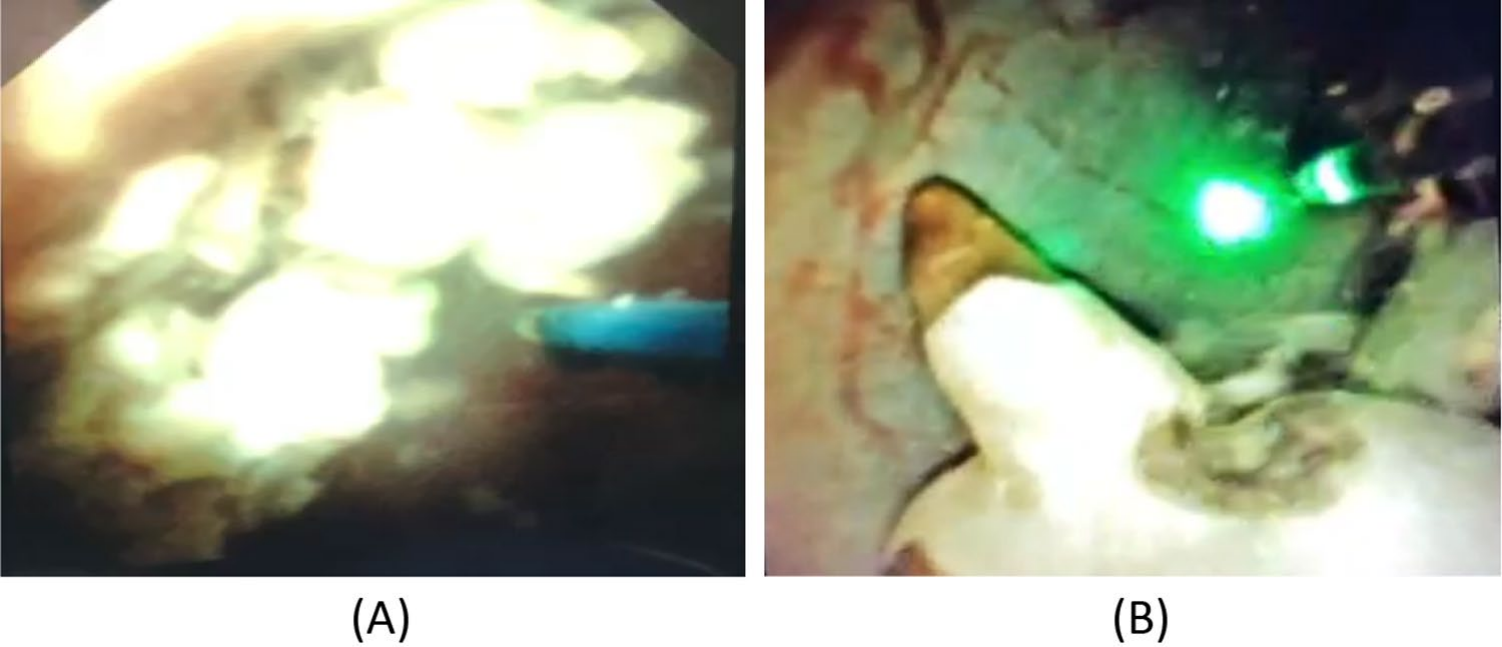
Image quality of Pusen Uscope (UE3011) (A) versus LithoVue FURS (B) (colour figure online)

Fever and hematuria were with higher incidence among Pusen group due to inferior image quality of Pusen device (Fig. 1) and in whom the access sheath was not used. The Pusen Uscope UE3011 is designed with a semiflexible shaft and a flexible tip only. We think this made Pusen FURS was more difficult in negotiation in pelvicalyceal system with more traumas to renal mucosa. Relative poor vision needs more wash for good vision which leads to increase in hematuria and intrarenal pressure with increase in incidence of UTI and fever. Immediate postoperative of 5-points Likert score survey had been sent to the surgeons to assess image quality between the two devices. Grading was on a scale from 1 to 5 corresponding to very poor to very good. Pusen group was 2 ± 1 and LithoVue group was 4 ± 1 that was significantly clear image with LithoVue group. This was parallel to Marchini et al., 2018 in which LithoVue had a higher resolution power than Pusen 3011 FURS [17]. Recently, Pusen has introduced a modified version (Pusen UE3022) with improved image quality and mechanical performance to overcome drawbacks of Pusen UE3011.

There is also one case with intraoperative ureteric mucosal injury caused by UAS which was managed by removal of access sheath and double J insertion (*p* = 0.1). Traxer and Thomas, 2013 demonstrated a large study of 359 cases of introduction of 12\14 Fr UAS, in which 167 cases had visible ureteric injury, 86% of them with low grade injury (injury of mucosal and not reach to smooth muscle), 10% with high grade injury (extending to smooth muscle) [18]. In The Clinical Research Office of the Endourological Society (CROES) URS Global Study, the complications among 11,885 worldwide patients who underwent URS are introduced. The most frequent complications were fever, bleeding and failed procedures. The overall complication rate was 7.4% which are comparable with our results [19].

Despite the effectiveness of FURS, the high costs hinder FURS being embraced worldwide, particularly in developing countries [20]. Variability in reusable FURS costs is determined by the initial purchase price, the cost of repair and the cost associated with sterile reprocessing whereas the cost of single use FURS is defined with the initial purchase price only [21]. Comparing baseline purchase price of both FURS provided by the manufacturer in our study was insignificant (*P* = 0.86) (Table 3).

We think that we are the first study in Egypt comparing these two types of FURS and we had several potential limitations that should be considered. First, sample size was relatively small. The studies with a small sample size were more likely to overestimate the treatment effect than those with larger sample sizes. Second, the mechanical and irrigation properties of both single use FURS were not investigated.

## Conclusion

RIRS can be performed effectively with Pusen 3011 and LithoVue single use flexible ureteroscopy in patients diagnosed with renal calculi less than 2 cm with superior outcomes with LithoVue.

## Data Availability

The datasets generated during and/or analysed during the current study are available from the corresponding author on reasonable request.

## Abbreviations

CT scan: Computerized tomography
FURS: Flexible ureteroscopy
PNL: Percutaneous nephrolithotomy
SWL: Extracorporeal shockwave lithotripsy
SFR: Stone free rate
US: Ultrasound
UAS: Ureteral access sheath
URS: Ureteroscopy
UTI: Urinary tract infection
